# HLA-related genetic susceptibility in autoimmune hepatitis according to autoantibody profile

**DOI:** 10.3389/fimmu.2022.1032591

**Published:** 2022-10-13

**Authors:** Eduardo Luiz Rachid Cancado, Juliana Goldbaum-Crescente, Debora Raquel B. Terrabuio

**Affiliations:** ^1^ Hospital das Clínicas da Faculdade de Medicina da Universidade de São Paulo, São Paulo, Brazil; ^2^ Instituto de Medicina Tropical da Faculdade de Medicina da Universidade de São Paulo, São Paulo, Brazil

**Keywords:** autoimmune hepatitis, autoantibodies, human leucocyte antigens (HLA), antismooth muscle antibodies, antinuclear antibodes, antiliver kidney type 1 microsome antibodies, antiliver cytosol type 1 antibodies, anti-soluble liver antigen antibodies

## Abstract

Although the prevalence of autoimmune hepatitis in first-degree relatives is small, the relationship between genetic markers, especially human leucocyte antigens (HLA), and susceptibility to this disease, has been studied for over three decades. The genetic susceptibility to AIH is believed to be different in the two subtypes of the disease, AIH type 1 and AIH type 2. Type 1 AIH has anti-smooth muscle and anti-nuclear antibodies as its main markers, while those of type 2 AIH are the anti-liver/kidney microsome type 1 and anti-liver cytosol type 1 antibodies. The anti-soluble liver antigen/liver-pancreas antibodies, which, in addition to being present in both subtypes, mark an important number of patients without serological markers. Therefore, a third type of disease is questionable. The vast majority of immunogenetic studies compare the differences between the two main types and make no difference between which antibodies are present to define the subtype. This review seeks to analyze what was most important published in the AIH in this context, trying to relate the HLA alleles according to the AIH marker autoantibodies.

## Introduction

Although autoimmune hepatitis (AIH) has very little familial aggregation, several markers of genetic susceptibility are investigated in an attempt to help understand the pathogenesis of the disease. The most studied markers, which demonstrated the greatest relationship between these variables, are undoubtedly the human histocompatibility antigen (HLA) alleles encoded by highly polymorphic genes linked to the major histocompatibility complex (MHC), on the short arm of chromosome 6. In addition to genetic markers, AIH has important serological autoantibodies that help in diagnosis, classification and, in a way, are related to the severity of the disease and the therapeutic response.

Several studies, over the decades, sought to report the types of AIH according to the genetic profile of HLA antigens. However, the geographic variation of these susceptibility markers is very large and this relationship with the classification of AIH has not been an easy task.

Following the revised diagnostic criteria of the international AIH group, from 1999, the reactivity of antinuclear antibodies (ANA), anti-smooth muscle antibodies (ASMA) and anti-liver kidney microsome antibodies type 1 (anti-LKM1) at titers >1/80, in adults, would be one of the most important criteria for the diagnosis. The presence of DRB1*03 or DRB1*04 should only be scored in case of autoantibody negativity. In these criteria, HLA reactivity was extended to other alleles, outside DR3 and DR4, according to geographic variations (1). This requirement was not strictly followed and in several publications, the HLA score was an additional parameter, regardless of the autoantibody reactivity (2).

While the serological markers of AIH were restricted to ASMA, ANA and anti-LKM1, the aggregation of HLA alleles according to autoantibody reactivity appeared to be a relatively easy job. However, after further investigation of the target antigens for the classic autoantibodies of the disease and the description of new serological markers, this correlation has become even more difficult than expected, since there are often two or even three simultaneously reactive autoantibodies that could have a different or similar relationship with the HLA alleles.

Most studies on AIH analyze the susceptibility relationship of HLA alleles to AIH subtypes and not to a specific autoantibody. (**Tables 1, 2 Supplementary Material**). They also look at the relationship between different clinical parameters, such as age at disease onset, IgG levels, therapeutic response, and prognosis. The aim of this review was to analyze the relationship between genetic markers of susceptibility linked to HLA according to AIH subtypes and with individual serological markers of the disease.

## Type 1 autoimmune hepatitis

Initially, studies of genetic susceptibility in AIH performed in European and North American Caucasian patients without distinctions regarding AIH subtypes. As in most of the studies, the patients had ANA and/or ASMA as markers, from the moment the reactivity of these autoantibodies began to be established, the results did not differ significantly for type 1 AIH. Thus, genetic susceptibility related to HLA alleles was associated with class II alleles, DRB1*03:01,*04:01, DRB3*01:01 that encode HLA molecules DR3, DR4 and DR52 respectively, in European and North American Caucasian populations (3, 4). When extending these studies to other Caucasian and non-Caucasian populations, for example, for mixed-race Mexican patients, susceptibility was associated with the DRB1*04:04 allele, although these results were not confirmed in a later study (5, 6). In Japanese, the susceptibility ratio was related to the alleles DRB1*04:05 and DQB1*DRB1*04:01, DQB1*04:01 haplotype in addition to the heterozygous genotype DR4/DR8 (7, 8).

In South America, the genetic predisposition to AIH acquired a different pattern. While in Argentina and Venezuela the primary association with DRB1*13:01 was observed in children (9, 10), in Brazil, although this primary association was stronger in younger patients, it was also observed in in all age groups (11). The frequency of DQB1*06 in these three South American countries, in linkage disequilibrium with DRB1*13, was also increased, but in Brazil, five patents with this DR allele carried different DQB1 alleles, conferring to DR13 higher odds ratio. In these three countries, DRB1*03 had a secondary association in patients who were negative for DRB1*13:01. Unlike Venezuela and Argentina, in Brazil this association was observed in patients who were positive for ASMA with specificity for F-actin. In Brazil, there was no association with the DRB1*04, but in Argentinean adult patients, a greater susceptibility was observed in those with DRB1*04:05. However, the ASMA and ANA reactivity was more frequently observed in children and adults with AIH respectively. Maybe this reactivity could also be related to those predisposing HLA alleles.

Although the susceptibility relationship with DRB1*03:01 and DRB1*04:01 was observed in North American and Europeans Caucasians with type 1 AIH, when analyzing whether this relationship held with ASMA or ANA reactivity, the results are, indeed, very contradictory. In 1993, results from North American patients revealed that ASMA reactivity and high ANA titers were found in patients who had DRB1*04. In 1996, there was no relationship between ASMA reactivity and HLA alleles, but when studying antibody reactivity against F actin, the relationship with HLA B*08 and DRB1*03:01 was verified in younger patients and with worse prognosis (4, 12). All patients who died with liver failure had reactivity for antibodies against microfilaments, as did almost all who required liver transplantation when compared with those antiactin negative/ANA positive patients. In other populations, a greater predisposition to AIH has also been reported in patients who carry the DRB1*13 allele, such as in Indian patients (13). North American patients with DRB1*13 as the sole marker of susceptibility, excluding patients DRB1*03 and DRB1*04, had a lower frequency of relapse and a higher occurrence of sustained remission after treatment withdrawal than patients with DRB1*03 as a single susceptibility marker (14). However, no relationship with serological markers was characterized in these studies.

In British Caucasian children, the DRB1*13 allele was more prevalent in children with type 1 AIH with biliary alterations, also named as having autoimmune sclerosing cholangitis in relation to healthy controls and type 2 AIH. However, when comparing these children with those with genuine type 1 AIH, who more frequently had DRB1*03, there was no significant difference in relation to DRB1*13. In this study, there was no stratification according to type 1 AIH serological markers, but the predominance of ASMA can be expected, since ANA reactivity alone in children is less frequent. In addition, the profile of autoantibodies in classical type 1 AIH and in its variant with more evident biliary injury was quite similar (15). Results from different pediatric centers of Europe, North and South America reinforced those obtained by family-based association studies in Canadian-French patients by the transmission disequilibrium test that susceptibility to type 1 AIH is linked to DRB1*03 and/or DRB1*13 (16).

The results of the association of the DRB1*04 allele with autoantibodies are also difficult to interpret. Initially, this class II allele was related to ASMA reactivity and high ANA titers. However, this relationship was no further confirmed in patients from the same population (12, 17). In Japanese patients, the DRB1*04:05/DQB1*04:01 haplotype was related to reactivity for ASMA (7). One of the difficulties in interpreting and studying the relationship between HLA alleles and ANA reactivity is due to the lack of characterization of ANA patterns in studies that evaluate this disease. The most common ANA patterns related to AIH are the homogeneous and the fine speckled. Despite the lack of studies analyzing this stratification, there are investigations in which the relationship between the HLA profile and reactivity for antibodies against specific nuclear antigens was analyzed. No susceptibility relationship was observed with anti-histone, anti-chromatin and anti-single-stranded DNA antibodies (18–20). The only antibody reactivity that was related to DRB1*04 was that to double-stranded anti-DNA searched by ELISA and indirect immunofluorescence (20).

On the other hand, there is also a protective relationship between a given HLA and susceptibility to the disease, although with less evident and less concordant results in different populations. This relationship with DRB1*13:02 deserves attention, because this allele differs from DRB1*13:01 by only one amino acid at position 86, glycine for valine respectively (7). It is possible that valine at position 86 of DRB1*13:01 may modulate the immune response to a putative auto-antigen. This protective action of the DRB1*13:02 was observed in Latin America and Japan and in several autoimmune diseases such as rheumatoid arthritis, primary biliary cholangitis, Graves’ disease, Hashimoto’s thyroiditis and psoriasis (21). Other alleles that were also discriminated as protective for the development of type 1 AIH were DQB1*04, DRB1*11 and the DRB1*15:01/DQB1*06:02 haplotype (7, 10, 22). This protective action was generally evaluated in relation to type 1 AIH and very little analyzed in type 2 AIH and, even in type 1 AIH, there is no assessment as to whether it would have any specificity for a particular autoantibody.

In immunogenetic studies, other HLA alleles were related to AIH susceptibility, including class I and class II antigens, usually not in isolation but as haplotypes, such as A*01-B*08-C*07-DRB3*01:01 (DRB1*52) whose alleles are in linkage disequilibrium with DRB1*03, just as the DRB4*01:03 is with DRB1*04:01. On the other hand, the DQA1*01:03 and DQB1*06:03 were also found to be in strong linkage disequilibrium with DRB1*13:01 according to the haplotype HLA-DRB1*13:01-DQA1*01:03-DQB1*06:03 (3, 8, 11, 22). However, there is, in general, no relationship of these haplotypes with a specific profile of autoantibodies.

## Type 2 autoimmune hepatitis

Few immunogenetic studies have focused on studying the relationship between HLA alleles and reactivity for type 2 AIH markers. The biggest obstacle in carrying out these studies is the low prevalence of this type of autoimmune in different geographic regions, for example, its rarity in North American studies. Unlike the AIH-1 markers, commercial kits with specific antigens for the determination of both, anti-LKM1 and anti-liver cytosol type 1, are commercially available and better homogenization of patients in relation to the autoantibody profile is possible.

In a 1997 study, when comparing German patients with type 2 AIH with North American controls and patients with type 1 AIH, it was observed a higher frequency of alleles DRB1*07, DRB1*15, DRB4*01 and DQB1*06 alleles (23). However, no results remained significant after the p correction. Until then, the immunogenetic studies performed did not suggest a susceptibility relationship of type 2 AI with MHC class II antigens (24). That same year, in Spanish patients, a higher frequency of DQB1*02 was found in carriers of type 2 AIH, an antigen that is in linkage disequilibrium with DRB1*07. The relationship with DRB1*07 was recorded more frequently in patients with Hepatitis C, who also had anti-LKM1 reactivity (25). This concomitance of hepatitis C with anti-LKM1 reactivity with DRB1*07 was also reported in Italians (26).

In 1999, in Brazilian patients, both alleles mentioned above plus DRB4 were significantly more frequent in patients with type 2 AIH, almost entirely with anti-LKM1 (25 of 28 patients). As two patients, who were DRB1*07 positive, were not DQB1*02, the Odds Ratio for DRB1*07 was higher suggesting that this allele was related to increased susceptibility (11). In a study with a greater number of patients with type 2 AIH and with better stratification of anti-LKM1 and anti-liver cytosol type 1 antibodies, Canadian and French Caucasian children presented the DQB1*02:01 allele as the main genetic determinant of susceptibility. The DRB1*03 allele was significantly increased among patients with simultaneous reactivity to anti-LKM1 and anti-liver cytosol type 1 antibodies, as well as those with isolated reactivity to anti-liver cytosol type 1 compared to those with isolated reactivity to anti-LKM1. The DRB1*07 was the most representative allele in patients with isolated reactivity to anti-LKM1 (27). All patients with DRB1*03 and DRB1*07 carried DQB1*02, and for this reason the latter HLA allele conferred the highest odds ratio. The result of this study corroborated the conclusion of a previous family-based association study by the transmission disequilibrium test from the same Canadian-French research center that type 2 AIH is associated with DQB1*02 (16). As these three alleles are present with strong linkage disequilibrium, the discordant results are understandable and demonstrate that there is a need for additional studies with a larger number of patients and with well-defined autoantibody markers. In patients outside Europe and North and South America, few publications, without stratifying the two different markers, recorded the susceptibility of type 2 AIH with the HLA alleles. In India, DRB1*14 have been linked to this AIH subtype analyzed in 13 patients (13).

## Autoimmune hepatitis with anti-soluble liver antigen/liver pancreas antibody reactivity

Some studies have linked anti-SLA/LP reactivity with genetic susceptibility to HLA alleles. In a meta-analysis study conducted in 2015, 195 patients with anti-SLA/LP were cataloged and the DRB1*03 allotype was positively associated with anti-SLA/LP reactivity (28). A bias that hinders the analysis of these results is the concomitance of the reactivity of this marker with ASMA, ANA and, to a lesser extent, with anti-LKM1/anti-liver cytosol type 1, which makes it difficult to interpret the relationship with DRB1*03. Another important factor that can make interpretation more difficult is the almost universal concomitant reactivity of anti-Ro52 with anti-SLA/LP (29). From publications on rheumatologic diseases, the correspondence of anti-Ro/SS-A with DRB1*03 has already been characterized (30). Patients with AIH and reactivity to anti-Ro52 and anti-SLA/LP antibodies had a higher frequency of DRB1*03 and a lower occurrence of DRB1*04 than patients with anti-Ro52 (29). However, in an analysis of Greek patients with AIH, when patients were stratified for anti-Ro52 and anti-SLA/LP reactivity, the frequency of DRB1*03 was higher only for patients positive for the former marker (31).

In a 2021 study, the immunogenetic analysis of 62 Chinese patients with AIH and anti-SLA/LP reactivity was compared with that of 500 healthy controls. A concomitant ANA reactivity was very high and, surprisingly, the class I alleles, HLA B*35:01 and C*08:01, were the only ones significantly more frequent in this set of patients (32). Other class I HLA alleles and class II alleles HLA-B*08:01, B*40:02, DRB1*04: 01, DRB1*04: 05, DRB1*14: 01 and DRB1*16: 02 also had a clear trend of greater frequency, while DRB1*15:01 of lower frequency, but did not reach statistical significance after Bonferroni’s correction, probably because of the small sample size. In our experience, anti-Ro52 is present in about 90% of patients with anti-SLA/LP reactivity, in 25% of patients with ASMA with F actin specificity without anti-SLA/LP reactivity and 23% of anti-LKM1 patients (unpublished data). Therefore, anti-Ro52 reactivity is yet another bias in the interpretation of the relationship of HLA alleles with autoantibody reactivity for not only patients with anti T SLA/LP reactivity but also for patients with type 1 and type 2 AIH.

## Final considerations

Few studies have studied the relationship between HLA alleles and the reactivity of AIH autoantibodies. In part, the obstacles faced in this regard are related to the genetic differences inherent in different populations. However, there are also shortcomings related to the methodology of the studies, such as the small number of patients, because it is a relatively uncommon disease. Another obstacle also lies in the patient selection criteria in relation to autoantibody reactivity. Comparison between the results of different cohorts of patients is greatly impaired as the frequency of autoantibody reactivity is extremely variable between series. For example, there are studies with a predominance of patients with reactivity for ANA and little reactivity for ASMA and vice versa. In other series, patients without autoantibody markers and patients with reactivity for antimitochondrial antibodies are included.

The ASMA reactivity may correspond to antibodies against antigens present on microfilaments, intermediate filaments, and microtubules, but the target antigen of this pattern in AIH is located in the polymerized form of actin (F-actin) of microfilaments and few studies adopt this criterion. Regarding ANA, little is known which would be their true target antigen(s) in AIH. Histones may potentially be the target antigen of ANA with the homogeneous pattern, but there are five types of histones and when testing the relationship between HLA alleles and anti-histone antibodies, no positive results were found. Regarding the fine speckled pattern of ANA, the target antigen is also still not identified. The rare studies that were more rigorous in selecting patients with ASMA/F-actin antibodies suggested a more important relationship with the DRB1*03 and DRB1*13 alleles. Regarding type 2 AIH, as the target antigens are better defined, the DRB1*07 and DRB1*03 alleles seem to be more related to anti-cytochrome P450 IID6 and anti-forminotransferase cyclodeaminase, respectively. Likewise, anti-SLA/LP, whose target antigen is the synthase converting O-phosphoseryl-tRNA to selenocysteinyl-tRNA, have a very close relationship with DRB1*03.

Celiac disease is a typical example of an autoimmune disease in which the genetic component (DQB1*02 and DQB1*08), the involved auto-antigen (tissue transglutaminase), the environmental trigger (gliadin) have been identified. Tissue transglutaminase-deaminated gliadin peptides that are recognized by DQ2/DQ8+ antigen presenting cells to T helper cells start the activation and maturation of B-lymphocytes that culminate in the production of anti-tissue transglutaminase antibodies. In addition, there is release of pro-inflammatory cytokines that, together with T killer cells, initiates the typical lesion of the intestinal mucosa (33). Studies in AIH are necessary with strict criteria in choosing patients with a well-defined diagnosis, who have a unified profile of autoantibodies that allows the characterization of specific target antigens. Perhaps this way we can identify, as in celiac disease, the environmental agent that triggers AIH and a more precise relationship between HLA alleles and autoantibody reactivity, finally developing specific curative treatment strategies.

## Author contributions

EC: manuscript design, reading the references, write the manuscript. JC: survey of bibliographic references, reading the references and obtaining data of relevance to the manuscript, revision. DT: survey of bibliographic references obtaining data of relevance to the manuscript, co-write the manuscript. All authors contributed to the article and approved the submitted version.

## Conflict of interest

The authors declare that the research was conducted in the absence of any commercial or financial relationships that could be construed as a potential conflict of interest.

## Publisher’s note

All claims expressed in this article are solely those of the authors and do not necessarily represent those of their affiliated organizations, or those of the publisher, the editors and the reviewers. Any product that may be evaluated in this article, or claim that may be made by its manufacturer, is not guaranteed or endorsed by the publisher.

## Abbreviations

HLA, human leucocyte antigen; AIH, autoimmune hepatitis; MHC, Major histocompatibility complex; ANA, antinuclear antibodies; ASMA, anti-smooth muscle antibodies; anti-LKM1, anti-liver kidney microsome type 1 antibodies; anti-SLA/LP, anti-soluble liver antigen/liver pancreas; SSA, syndrome Sjögren antigen.
