# Supplementary material for: HLA-related genetic susceptibility in autoimmune hepatitis according to autoantibody profile
Source: Front Immunol. 2022 Oct 13;13:1032591. doi: 10.3389/fimmu.2022.1032591 (PMC9606223; doi:10.3389/fimmu.2022.1032591)
Supplement: Supplementary file 1 [file Table_1.docx]

Table 1. Most important data from publications on the relationship between frequency of HLA alleles and characteristics of patients with type 1 AIH

| Authors | Autoantibody | Technique HLA typing | HLA class I associations | | HLA class II associations | Results |
| --- | --- | --- | --- | --- | --- | --- |
| Donaldson PT^1^, 1991; 96 British patients; 100 HC | AASMA/ANA | Complement- dependent microcytotoxicity assay; DR typing: “panning” technique and immunomagnetic beads | A*01/B*08/DRB1*03 | | | Susceptibility to AIH  Younger age; more relapse and liver transplantation |
|  |  |  |  | | DRB1*04/DR*03 negative | Secondary association |
| Czaja AJ^2^ 1993, 122 North American patients with AIH; 63 patients with viral hepatitis | ASMA/ANA | Microlymphocytotoxicity technique, and multiple locally derived and appropriately validated sera and commercially available sera were used to define each specificity | A*01/B*08/DRB1*03 | | | Predisposing association |
|  |  |  |  | | DRB1*04 | ASMA reactivity  High titers of ANA reactivity  Concurrent autoimmune diseases |
| Fainboim L^3^, 1994; 52 Caucasoid Argentinean patients 197 HC | ASMA, ANA ≥ 1/80 | Complement-dependent microcytotoxicity using sera from the 10th and 11^th^ International Histocompatibility Workshops, supplemented with local and commercial sera; HLA-DR and -DQ typing was performed with HLA class-II-positive cells isolated by immunomagnetic beads) | No associations | | DRB1*06 (31 out of 32 patients were DRB1*13:01 | Predisposing association |
|  |  |  |  |  | DQB1*06:03 | Predisposing association |
| Czaja AJ^4^, 1995  65 North American patients | ANA (anti-histone) | Microlymphocytotoxicity technique was used and multiple locally derived and commercially available sera were employed to define each specificity | All patients  No associations | | 54 patients  No associations | Do not have clinical relevance |
| Czaja AJ^5^, 1996  95 North American Caucasoid patients  80 HC | 73 patients with  AAA | Microlymphocytotoxicity and restriction fragment length polymorphism | 90 patients  79 HC  B*08 | | 75 patients  All HC  DRB1*DR3 | Younger patients  Poor prognosis |
|  | ASMA |  | No associations | | No associations | Do not have clinical relevance |
| Czaja AJ^6^, 1997  53 North American Caucasoid patients; 80 HC | Anti-ds-DNA (ANA)  ELISA/IIF | Microlymphocytotoxicity and restriction length polymorphism | All patients  79 HC  No associations | | 45 patients  All HC  DR4 | ELISA positive patients had poorer response to therapy; adjustments of therapy |
|  | Anti-ss-DNA |  | No associations | | No associations | Do not have clinical relevance |
| Czaja AJ^7^, 1997  86 North American Caucasoid patients; 102 HC* | ASMA/ANA | Microlymphocytotoxicity (Class I)  PCR-SSP (class II) | 84 patients; 98 HC  No associations | | DRB4*01:03 | > concurrent autoimmune disease |
|  |  |  |  |  | DRB1*0301  DRB1*03:01/DRB3*01:01 | patients with a poor treatment response |
|  |  |  |  |  | DRB1*04:01; DRB1*04:01/DRB4*01:03 | Lower frequency of hepatic death or transplantation |
|  |  |  |  |  | DRB1*03:01 | Younger than DRB1*0401 |
|  |  |  |  |  | Number of lysine at position 71 | Treatment outcome may relate to the number of lysine copies that are encoded at DR𝞫71. |
| Vazquez-Garcia MN^8^, 1998  30 Mexican Mestizo patients; 175 HC | ASMA/ANA | Microlymphocytotoxicity (Class I)  PCR-SSO, PCR-SSP (class II) | No associations | | DRB1*04:04 | Predisposing association and late onset |
|  |  |  |  |  | DQA1*05:01 | Predisposing association and early onset |
|  |  |  |  |  | DQB1*03:01 | Protection from AIH |
| Bittencourt P^9^, 1999  111 Brazilian patients;  Highly admixed population  129 HC | ASMA (91 patients with AAA)  ANA | PCR-SSP | Not performed | | DRB1*13 | Primary association  More evident in younger patients |
|  |  |  |  |  | DRB1*03/DRB1*13 negative | Secondary association |
| Pando M^10^, 1999  122 pediatric Argentinean Caucasoid patients; 208 HC | ASMA(AAA) ANA | High resolution oligonucleotide typing | Not performed | | DRB1*13:01 | Primary association  lower therapeutic response rate |
|  |  |  |  |  | DRB1*03:01/DRB1*13:01 negative | Secondary association |
|  |  |  |  |  | DRB1*13:02 | Protection from AIH |
| Pando M^10^, 1999  84 adult Argentinean Caucasoid patients; 208 HC | ANA > ASMA/AAA | High resolution oligonucleotide typing |  | | DRB1*04:05 | Predisposing association |
|  |  |  | A*11/DRB1*0405 | | | Extra-hepatic autoimmune manifestations |
| Chen L^11^, 2000  36 Japanese patients; 74 HC | anti-chromatin antibodies | Standard Microlymphocytotoxicity | No association | | No association | Do not have clinical relevance |
| Goldberg A^12^  2001  39 Brazilian patients  22 HC (Highly admixed population) | ASMA/AAA | HLA-DRB1*13 sequencing | Not performed | | DRB1*13:01 |  |
| Qiu DK^13^, 2003  32 chinese patients; 48 HC | ASMA/ANA | Sequence specific primers (PCR-SSP) | Not performed | | DRB1*04  Trends DRB1*04:05 | Not determined |
| Czaja AJ^14^, 2003  142 North American patients; 102 HC | Anti-chromatin antibodies | Microlymphocytotoxicity; restriction fragment length polymorphism, or PCR-SSP | Not performed | | No association | Disease activity |
| Muratori P, 2005^15^;57 Italians | ASMA/ANA/without markers | Microlymphocytotoxicity and PCR-SSP | B*08-C*07-DRB1*03 | | | Predisposing association |
|  |  |  |  | | DRB3 | higher levels of ALT at baseline |
|  |  |  |  | | DRB1*11 (less commonly) | Protection from AIH |
| Shankarkumar U^16^, 2005  20 Western Indian patients;120 HC | Not determined | Microlymphocytotoxicity, PCR-SSP, PCR-SSOP | A*02:22A*32:01 A*68:01:02 B*35 B*55:01, Cw*01:02 Cw*07:01:01 | | DRB1*03:01, DRB1*13:01, DRB1*14 DRB1*15:01 | Increased among AIH patients when compared with the HC |
|  |  |  | A*02:11, A*33:03, B*40:06, Cw*15:02:01 | |  | Significantly reduced among AIH patients. |
| Fortes MP^17^, 2007  41 Venezuelan mestizo patients  111 HC | ASMA/ANA | PCR-SSP |  | | DRB1*13:01  DRB1*03:01/DRB1*13:01 negative | Primary predisposing association; more evident in younger patients |
|  |  |  |  |  | DRB1*13:01 | High propensity towards developing cirrhosis |
|  |  |  |  |  | DQB1*04 | Protection from AIH |
|  |  |  |  |  | DRB3 | Secondary predisposing association |
|  |  |  | A*01-B*08-DQB1*02-DRB1*03- DRB3; DQB1*05-DRB1*13:01; DQB1*06-DRB1*13:01; A*02-DRB1*13:01, B*45-DRB3 | | | Predisposing association |
| Miyake Y^18^, 2008,  79 Japanese patients | ASMA/ANA | PCR- SSO hybridization | Not performed | | DRB1*04 | Tendency of higher levels of immunoglobulin  > 30 years,  Thyroid diseases |
|  |  |  |  |  | DRB1*02 | Lower frequency of concurrent autoimmune disease |
|  |  |  |  |  | Neither DRB1*04 nor DRB1*02  No patients with DRB1*03 | Lower levels of immunoglobulin |
| Lim YS^19^ 2008; 62 Korean patients, 154 HC | ASMA/ANA | PCR–sequence based typing (PCR–SBT) | No associations | | DRB1*04:05; DQB1*04:01 | Predisposing association |
| Ngu JH^20^, 2013  77 New Zealand Caucasians. 485 HC | ASMA/ANA | Predesigned TaqMan SNP genotyping assays following the manufacturer’s instructions | Not performed | | DRB1*03:01 | The C FAS allele associated with a more aggressive disease phenotype. |
| Hassan N^21^, 2013; 58 pediatric Pakistani patients; 912 HC | ASMA/ANA and anti-LKM1 | NIH Microlymphocytotoxicity assay using eosin dye extrusion (class I)  PCR/SSP (class II) | A*02; A*09 A*10, A*19, B*15, B*40 | | DR6 (DRB1*13); DRB1*14 (p = 0.017) | Predisposing association |
| Umemura T^22^,  2014  156 Japanese patients  201 HC | ASMA/ANA | Luminex multi-analyzer profiling system with a LAB type SSO One Lambda typing kit (One Lambda, Inc. Canoga Park, CA). HLA genotypes were determined by sequence-based typing, | A*24:02 C*01:02 | | DRB1*04:05/DQB1*04:01 | Elevated serum IgG and ASMA positivity |
|  |  |  |  | | DRB1*15:01-DQB1*06:02 haplotype | Protection from AIH; development of hepatocarcinoma |
|  |  |  |  | | DRB1*08:03-DQB1*06:01 | Development of hepatic failure |
| Kaur N^23^, 2014  55 North Indian patients  128 HC | ASMA/ANA | PCR-SSP using low resolution HLA typing kits | No associations | | DRB1*04  DRB1*13 | In pediatric population |
|  |  |  |  |  | DRB1*08 | In adult population |
| Furumoto Y^24^, 2015  132 Japanese AIH patients  31973 HC | ANA, AMA | PCR-based reverse sequence specific oligonucleotide typing | Not performed | | DRB1*04 | Higher levels of IgG and IgM (older patients), younger than DR4 negative patients |
| Baharlou R^25^, 2016  45 Iranian AIH. 100 HC | ASMA/ANA | PCR/SSP | Not performed | | DRB1*03, DRB1*04, DRB1*08, DRB1*13 | Predisposing association |
|  |  |  |  |  | HLA-DRB1*11, HLA-DRB1*15, HLA-DRB5 | Protection from AIH |
| Oka S^26^, 2017  360 Japanese patients  1026 HC | ASMA/ANA | PCR-SSO | Not performed | | DRB1*04:01  DRB1*04:05  DQB1*04:01  DRB1*04/DRB1*08 heterozygous | Predisposing association |
|  |  |  |  |  | DRB1*04:05-DQB1*04:01/DRB1*08:02-DQB1*03:02 DRB1*04:05-DQB1*04:01/DRB1*08:03-DQB1*06:01 | Susceptible diplotypes |
|  |  |  |  |  | DRB1*04:05 | Higher serum levels of IgG and IgM, International AIH Hepatitis Group score, positive ASMA, and the rate of definite AIH |
|  |  |  |  |  | DRB1*13:02 | Protective association |
| [Mendoza-Carrera](https://pubmed.ncbi.nlm.nih.gov/?size=200&term=Mendoza-Carrera+F&cauthor_id=31570816) F^27^, 2019  56 Mexican patients | ANA/ASMA | sequencing of exon 2 of the HLA–DRB1 gene SSO | No associations | | | Do not have clinical relevance |
| Ma Y^28^, 2021  177 children with European Ancestry (100 AIH-1, 77 Autoimmune sclerosing cholangitis)  209 European HC | ASMA/ANA | PCR/sequence specific primers (SSP) | B*08; DRB1*03; A*01/B*08/DRB1*3 | | | Susceptibility to AIH-1, AIH-2 and ASC |
|  |  |  |  | Homozygosis for DRB1*03 | | The strongest risk factor; The highest ANA titers |
|  |  |  |  | HLA DRB1*04  DRB1*15 (DR15) | | Protection from AIH-1 e ASC |
|  |  |  |  | DRB1*13 | | Susceptibility to ASC > HC, AIH-2 |
|  |  |  |  | Homozygous DRB1*03 or DRB1*13 | | > fibrosis at disease onset |
|  |  |  |  | DRB1*03 or *DRB1*13 in addition to DRB1*07 | | > severe disease in AIH-1, and ASC |

AAA: antiactin antibodies; AIH: autoimmune hepatitis; AMA: antimitochondrial antibodies; ANA: antinuclear antibodies; ASC: autoimmune sclerosing cholangitis; ASMA: antismooth muscle antibodies; ds-DNA: double strand DNA; HC: healthy controls; PCR: polymerase chain reaction; sd-DNA: single strand DNA; SSP: Sequence specific primers; SSO: Sequence specific oligonucleotides

References

1. [Donaldson](https://pubmed.ncbi.nlm.nih.gov/?size=200&term=Donaldson+PT&cauthor_id=2010165) PT, [Doherty](https://pubmed.ncbi.nlm.nih.gov/?size=200&term=Doherty+DG&cauthor_id=2010165) DG, [Hayllar](https://pubmed.ncbi.nlm.nih.gov/?size=200&term=Hayllar+KM&cauthor_id=2010165) KM, [McFarlane](https://pubmed.ncbi.nlm.nih.gov/?size=200&term=McFarlane+IG&cauthor_id=2010165) IG, [Johnson](https://pubmed.ncbi.nlm.nih.gov/?size=200&term=Johnson+PJ&cauthor_id=2010165) PJ, [Williams](https://pubmed.ncbi.nlm.nih.gov/?size=200&term=Williams+R&cauthor_id=2010165) R. Susceptibility to autoimmune chronic active hepatitis: human leukocyte antigens DR4 and A1-B8-DR3 are independent risk factors. Hepatology (1991) 13:701-6.
2. [Czaja](https://pubmed.ncbi.nlm.nih.gov/?size=200&term=Czaja+AJ&cauthor_id=8224654) AJ, [Carpenter](https://pubmed.ncbi.nlm.nih.gov/?size=200&term=Carpenter+HA&cauthor_id=8224654) HA, [Santrach](https://pubmed.ncbi.nlm.nih.gov/?size=200&term=Santrach+PJ&cauthor_id=8224654) PJ, [Moore](https://pubmed.ncbi.nlm.nih.gov/?size=200&term=Moore+SB&cauthor_id=8224654)SB. Significance of HLA DR4 in type 1 autoimmune hepatitis. Gastroenterology (1993); 105:1502-7. doi: 10.1016/0016-5085(93)90157-8.
3. Fainboim L, Marcos Y, Pando M, Capucchio M, Reyes GB, Galoppo C, et al. [Chronic active autoimmune hepatitis in children. Strong association with a particular HLA-DR6 (DRB1*1301) haplotype.](https://pubmed.ncbi.nlm.nih.gov/7860360/) Hum Immunol (1994) 41:146-50. doi: 10.1016/0198-8859(94)90008-6.
4. Czaja AJ, Ming C, Shirai M, Nishioka M. Frequency and significance of antibodies to histones in autoimmune hepatitis. J Hepatol (1995) 23:32-8. doi: 10.1016/0168-8278(95)80308-4.
5. Czaja AJ, Cassani F, Cataleta M, Valentini P, Bianchi FB. Frequency and Significance of Antibodies to Actin in Type 1 Autoimmune Hepatitis. Hepatology. (1996) 24:1068-73. doi: 10.1002/hep.510240515.
6. Czaja A, Morshed SA, Parveen S, Nishioka M. Antibodies to single-stranded and double-stranded DNA in antinuclear antibody-positive type 1-autoimmune hepatitis. Hepatology (1997) 26:567-72. doi: 10.1002/hep.510260306.
7. Czaja AJ, Strettell MD, Thomson LJ, Santrach PJ, Moore SB, Donaldson PT, Williams R. [Associations between alleles of the major histocompatibility complex and type 1 autoimmune hepatitis.](https://pubmed.ncbi.nlm.nih.gov/9021941/) Hepatology (1997);25:317-23. doi: 10.1002/hep.510250211.
8. [Vazquez-García](https://pubmed.ncbi.nlm.nih.gov/?size=200&term=V%C3%A1zquez-Garc%C3%ADa+MN&cauthor_id=9672174) MN, [Alaez](https://pubmed.ncbi.nlm.nih.gov/?size=200&term=Al%C3%A1ez+C&cauthor_id=9672174) C, [Olivo](https://pubmed.ncbi.nlm.nih.gov/?size=200&term=Olivo+A&cauthor_id=9672174) A, [Debaz](https://pubmed.ncbi.nlm.nih.gov/?size=200&term=Debaz+H&cauthor_id=9672174) H, [Perez-Luque](https://pubmed.ncbi.nlm.nih.gov/?size=200&term=P%C3%A9rez-Luque+E&cauthor_id=9672174) E, [Burguete](https://pubmed.ncbi.nlm.nih.gov/?size=200&term=Burguete+A&cauthor_id=9672174) A,et al. MHC class II sequences of susceptibility and protection in Mexicans with autoimmune hepatites. J Hepatol (1998) 28:985-90. doi: 10.1016/s0168-8278(98)80347-4.
9. Bittencourt PL, Goldberg AC, Cançado ELR, Porta G, Carrilho FJ, Farias AQ, et al. Genetic Heterogeneity in Susceptibility to Autoimmune Hepatitis Types 1 and 2. Am J Gastroenterol 1999; 94:1906-13. doi: 10.1111/j.1572-0241.1999.01229.x
10. Pando M, Larriba J, Fernandez GC, Fainboim H, Ciocca M, Ramonet M, et al. Pediatric and Adult Forms of Type I Autoimmune Hepatitis in Argentina: Evidence for Differential Genetic Predisposition. Hepatology (1999) 30:1374-80. doi: 10.1002/hep.510300611.
11. Li L, Chen M, Huang DY, Nishioka M. Frequency and significance of antibodies to chromatin in autoimmune hepatitis type I. J Gastroenterol Hepatol (2000) 15:1176-82. doi: 10.1046/j.1440-1746.2000.02325.x.
12. Goldberg AC, Bittencourt PL, Mougin B, Cancado EL, Porta G, Carrilho JF, et al. Analysis of HLA Haplotypes in Autoimmune Hepatitis Type 1: Identifying the Major Susceptibility Locus. Hum Immunol (2001) 62:165-9. doi: 10.1016/s0198-8859(00)00234-2.
13. Qiu DK, Ma X.J. [Relationship between human leukocyte antigen-DRB1 and autoimmune hepatitis type I in Chinese patients.](https://pubmed.ncbi.nlm.nih.gov/12519226/) Gastroenterol Hepatol. (2003) 18:63-7. doi: 10.1046/j.1440-1746.2003.02918.x.
14. [Frequency and significance of antibodies to chromatin in autoimmune hepatitis.](https://pubmed.ncbi.nlm.nih.gov/12924665/) Czaja AJ, Shums Z, Binder WL, Lewis SJ, Nelson VJ, Norman GL. Dig Dis Sci. (2003) ;48:1658-64. doi: 10.1023/a:1024748714580.
15. Muratori P, Czaja AJ, Muratori L, Pappas G, Maccariello S, Cassani F, et al.. Genetic distinctions between autoimmune hepatitis in Italy and North America. World J Gastroenterol (2005) 11:1862-6. doi: 10.3748/wjg.v11.i12.1862.
16. Shankarkumar U, Amarapurkar DN, Kankonkar S. Human leukocyte antigen allele associations in type-1 autoimmune hepatitis patients from western India. J Gastroenterol Hepatol (2005); 20:193-7. doi: 10.1111/j.1440-1746.2004.03608.x.
17. Fortes MP, Machado IV, Gil G, Fernandez-Mestre M, Dagher L, Leon RV , et al. Genetic contribution of major histocompatibility complex class II region to type1 autoimmune hepatitis susceptibility in Venezuela. Liver Int (2007) 27:1409-16. doi: 10.1111/j.1478-3231.2007.01581.x.
18. Miyake Y, Iwasaki, et al. Y, Takaki A, Onishi T, Okamoto R, Takaguchi KHuman leukocyte antigen DR status and clinical features in Japanese patients with type 1 autoimmune hepatitis. Hepatol Res 2008 ;38:96-102. doi: 10.1111/j.1872-034X.2007.00204.x
19. Lim YS, Oh HB, Choi SE, Kwon OJ, Heo YS, Lee HC, et al. Susceptibility to type 1 autoimmune hepatitis is associated with shared amino acid sequences at positions 70–74 of the HLA-DRB1 molecule. J Hepatol (2008); 48(1):133–9. https://doi.org/10.1016/j.jhep.2007.08.019 PMID: 18022727.
20. Ngu JH, Wallace MC, Merriman TR, Gearry RB, Stedman CA, Roberts RL, Association of the HLA locus and TNF with type I autoimmune hepatitis susceptibility in New Zealand Caucasians. Springerplus (2013) 2:355. doi: 10.1186/2193-1801-2-355.
21. Hassan N, Siddiqui AR, Abbas Z, Hassan SM, Soomro GB, Mubarak M, et al. Clinical Profile and HLA Typing of Autoimmune Hepatitis From Pakistan. Hepat Mon 2013;13(12):e13598. doi: 10.5812/hepatmon.13598.
22. Umemura T, Katsuyama Y, Yoshizawa K, Kimura T, Joshita S, Komatsu M, et al, Human Leukocyte Antigen Class II Haplotypes Affect Clinical Characteristics and Progression of Type 1 Autoimmune Hepatitis in Japan.. PLoS One (2014) ;9(6):e100565. doi: 10.1371/journal.pone.0100565
23. Kaur N, Minz RW, Anand S, Saikia B, Aggarwal R, Das A, et al. HLA DRB1 Alleles Discriminate the Manifestation of Autoimmune Hepatitis as Type 1 or Type 2 in North Indian Population, J Clin Exp Hepatol (2014) 4:14-8. doi: 10.1016/j.jceh.2013.12.002.
24. Furumoto Y, Asano T, Sugita T, Abe H, Chuganji Y, Fujiki K et al. Evaluation of the role of HLA-DR antigens in Japanese type 1 autoimmune hepatitis. BMC Gastroenterol (2015)15:144. doi: 10.1186/s12876-015-0360-9.
25. Baharlou R, Faghihi-Kashani A, Faraji F, Najafi-Samei M, Setareh M, Zamani F, et al. HLA-DRB1 alleles of susceptibility and protection in Iranians with autoimmune hepatitis. Hum Immunol (2016) 77:330-5. doi: 10.1016/j.humimm.2016.01.007.
26. Oka S, Furukawa H, Yasunami M, Kawasaki A, Nakamura H, Nakamura M, et al. HLA-DRB1 and DQB1 alleles in Japanese type 1 autoimmune hepatitis: The predisposing role of the DR4/DR8 heterozygous genotype. PLoS One (2017);12(10):e0187325. doi: 10.1371/journal.pone.0187325.
27. Mendoza-Carrera F, Gastelum-Meza MA, Ramirez-Garcia J, Davalos-Cobian C, Castro-Martinez XH. No association of HLA-DRB1 and TNF alleles in Mexican patients with autoimmune hepatites. Genes Immun (2019) 20:678-83. doi: 10.1038/s41435-019-0086-8.
28. Ma Y, Su H, Yuksel M, Longhi MS, McPhail MJ, Wang P, et al. Human Leukocyte Antigen Profile Predicts Severity of Autoimmune Liver Disease in Children of European Ancestry. Hepatology (2021) 74:2032-2046. doi: 10.1002/hep.31893.
