# Supplementary material for: HLA-related genetic susceptibility in autoimmune hepatitis according to autoantibody profile
Source: Front Immunol. 2022 Oct 13;13:1032591. doi: 10.3389/fimmu.2022.1032591 (PMC9606223; doi:10.3389/fimmu.2022.1032591)
Supplement: Supplementary file 2 [file Table_2.docx]

Table 2. Most important data from publications on the relationship between frequency of HLA alleles and characteristics of patients with type 2 autoimmune hepatitis, and with reactivity to anti-SLA/LP

| Authors | Autoantibody | HLA typing technique | HLA class I associations | HLA class II associations | Results |
| --- | --- | --- | --- | --- | --- |
| Jurado A, 1997  15 Spanish patients;  60 HC | Anti-LKM1 | lymphocytotoxicity test for class 1; a reverse hybridization-based test for DRB1 typing and PCR-SSO for DQB1 typing | No associations | DQB1*02 | Risk factor for AIH-2 |
| Bittencourt P, 1999  28 Brazilian patients;  Highly admixed population  129 HC | Anti-LKM1 | PCR-PCR-SSP | Not performed | DRB1*07; DRB4*01; DQB1*02 | Risk factor for AIH-2 |
|  |  |  |  | DRB1*03/DRB1*07 negative | Risk factor for AIH-2 |
| Muratori P, 2005  17 Italians patients  372 HC | Anti-LKM1/Anti-LC1 | Microlymphocytotoxicity and Sequence specific primers (PCR-SSP) | No associations | No associations | Risk factors for AIH occur similarly in Italian patients with type 1 and type 2 AIH |
|  |  |  |  | DRB1*07 | > AIH-1 (not significant) |
| 2006, Djilali-Saiah I  60 Caucasian Patients (10 Canadian, 50 French patients)  313 HC (213 French, 100 French Canadian) | Anti-LKM1 and Anti-LC1 | PCR-SSP/PCR-SSO | Not performed | DQB1*02:01 | primary genetic determinant of susceptibility |
|  |  |  |  | DRB1*03 | significantly increased among patients with both anti-LKM1 and anti-LC1 autoantibodies as well as in those with only anti-LC1 positive |
|  |  |  |  | DRB1*07 | Significantly associated with anti-LKM1+ alone |
|  |  |  |  | DRB1:03-DQB1*02:01+ve and DRB1*07- DQB1*02:01+ve patients | ALT values were significantly higher |
| Kaur N, 2014  13 North Indian patients  128 HC | Anti-LKM1 | PCR-SSP using low resolution HLA typing kits | No associations | DRB1*14 | Predisposing condition |
| Baharlou R, 20016  9 Iranian AIH. 100 HC | Anti-LKM1 | PCR/SSP | Not performed | DRB1*07, DRB1*13. | Predisposing condition |
|  |  |  |  | DRB1*03; Homozygosis for DRB1*03 represented the strongest risk factor | Susceptibility to AIH-2 > HC |
|  |  |  |  | A1-B8-DRB1*03 haplotype | Susceptibility to AIH-2 > HC |
|  |  |  |  | DRB1*07 | Susceptibility to AIH-2 > HC. AIH-1 and ASC |
|  |  |  |  | DRB1*07 in DRB1*03 negative | Susceptibility to AIH-2 > HC. AIH-1 and ASC; the highest risk |
|  |  |  |  | DQB1*02:01 | > HC |
|  |  |  |  | DRB1*15 | Protection |
|  |  |  |  | Homozygous DRB1*03 or DRB1*13 | associated with fibrosis at disease onset |
|  |  |  |  | DRB1*03 or DRB1*13 +/ DRB1*07 + | Associated with a more severe disease |
| Montano-Loza A, 2012  142 North American patients with AIH-1 | ASMA, ANA, anti-SLA/LP, anti-Ro52 | Restriction fragment length polymorphism (89 patients) or PCR-SSP (53 patients) | Not performed | Only the frequencies of DRB1*03 e *04 were analyzed. |  |
|  |  |  |  | DRB1*03 | Higher frequency |
|  |  |  |  | DRB1*04 | Lower frequency |
| Zhang HP, 2021  62 Chinese patients with anti-SLA/LP reactivity  500 HC | ANA,ASMA, | HLA class Ι (A, B, and C) and class II (DRB1 and DQB1) allelic genotypes PCR-SSP | B*35:01; C*08:01 |  | Predisposing condition |
|  |  |  | HLA-B*08:01, B*40:02 | DRB1*04:01, DRB1*04:05, DRB1*14:01, DRB1*16:02 | Predisposing condition, but lost significance after Bonferroni’s correction |
|  |  |  |  | DRB1*15:01 | Protection from AIH, but lost significance after Bonferroni’s correction |
|  |  |  |  | DRB1*04:05, DQB1*04:01 | Concurrent autoimmune disease; decompensated disease, but lost significance after Bonferroni’s correction |

AIH: autoimmune hepatitis; AIH-2: type 2 autoimmune hepatitis ; Anti-LKM1: anti-liver kidney microsome antibodies; Anti-LC1: anti-liver cytosol antibodies; anti-SLA/LP: anti-soluble liver antigen/liver pancreas; HC: healthy controls; PCR: polymerase chain reaction; SSP: Sequence specific primers; SSO: Sequence specific oligonucleotides

References

1. Jurado A, Cárdaba B, Jara P, Cuadrado P, Hierro L, de Andrés B, et al. [Autoimmune hepatitis type 2 and hepatitis C virus infection: study of HLA antigens.](https://pubmed.ncbi.nlm.nih.gov/9186828/) J Hepatol (1997) 26:983-91. doi: 10.1016/s0168-8278(97)80106-7.
2. Bittencourt PL, Goldberg AC, Cançado ELR, Porta G, Carrilho FJ, Farias AQ, et al. Genetic Heterogeneity in Susceptibility to Autoimmune Hepatitis Types 1 and 2. Am J Gastroenterol 1999; 94:1906-13. doi: 10.1111/j.1572-0241.1999.01229.x

Muratori P, Czaja AJ, Muratori L, Pappas G, Maccariello S, Cassani F, et al.. Genetic distinctions between autoimmune hepatitis in Italy and North America. World J Gastroenterol (2005) 11:1862-6. doi: 10.3748/wjg.v11.i12.1862.

1. Djilali-Saiah I, Fakhfakh A, Louafi H, Caillat-Zucman S, Debray D, Alvarez F. HLA Class II influences humoral autoimmunity in patients with type 2 autoimmune hepatitis. J Hepatol (2006) 45:844-50. doi: 10.1016/j.jhep.2006.07.034.
2. Kaur N, Minz RW, Anand S, Saikia B, Aggarwal R, Das A, et al. DRB1 Alleles Discriminate the Manifestation of Autoimmune Hepatitis as Type 1 or Type 2 in North Indian Population. J Clin Exp Hepatol. (2014) 4:14-8. doi: 10.1016/j.jceh.2013.12.002.
3. Ma Y, Su H, Yuksel M, Longhi MS, McPhail MJ, Wang P, et al. Human Leukocyte Antigen Profile Predicts Severity of Autoimmune Liver Disease in Children of European Ancestry. Hepatology (2021) 74:2032-2046. doi: 10.1002/hep.31893.
4. Montano-Loza AJ, Shums Z, Norman GL, Czaja AJ. Prognostic implications of antibodies to Ro/SSA and soluble liver antigen in type1 autoimmune hepatitis. Liver Int (2012) 32:85-92. doi: 10.1111/j.1478-3231.2011.02502.x.
5. Zhang HP, Liu YM, Li Z, Ma YX, Li LJ, Zhao DT et al. Clinical characteristics and HLA genotypes in Chinese patients with anti-SLA/LP-positive autoimmune hepatitis. Ann Transl Med (2021) 9:153. doi: 10.21037/atm-20-8036.
